# A Deep Learning-Enhanced Adaptive Kalman Filter with Multi-Scale Temporal Attention for Airborne Gravity Denoising

**DOI:** 10.3390/s26072216

**Published:** 2026-04-03

**Authors:** Lili Li, Junxiang Liu, Guoqing Ma, Zhexin Jiang

**Affiliations:** State Key Laboratory of Deep Earth Exploration and Imaging, College of Geoexploration Science and Technology, Jilin University, Changchun 130026, China; lilili@jlu.edu.cn (L.L.); junxiang23@mails.jlu.edu.cn (J.L.); jiangzx21@mails.jlu.edu.cn (Z.J.)

**Keywords:** airborne gravity data, adaptive Kalman Filter, multi-scale CNN, Bi-LSTM

## Abstract

**Highlights:**

**What are the main findings?**

- Airborne gravity data accuracy is limited by measurement conditions and requires noise filtering.
- Neural network-based Kalman filtering avoids manual parameter setting and enhances filtering accuracy.

**What are the implications of the main findings?**

- Enhanced airborne gravity data quality supports high-precision geophysical exploration.
- The proposed method provides a reliable technical reference for similar remote sensing data processing tasks.

**Abstract:**

Airborne gravity survey serves as a rapid remote sensing technique for mapping subsurface mineral target and geological structure over large areas. The raw gravity data contains significant noise corrupted by airflow and the flight platform’s attitude. The Kalman Filter (KF) is an effective method for airborne gravity data denoising, but its processing accuracy is highly dependent on the empirical parameters. The multi-scale CNN-LSTM-attention adaptive Kalman Filter (MSC-LA-AKF) method is proposed to obtain high precision gravity data, which combines the multi-scale CNN (MSC), bidirectional long short-term memory (Bi-LSTM) and attention mechanism for adaptively estimating the parameters of KF. The multi-scale CNN uses convolution kernel of varying sizes to extract signal features at different scales. The Bi-LSTM combines two LSTM layers in opposite directions to extract the signal features at bidirectional time series, and can effectively identify time-varying noise signals. A multi-head attention mechanism with four attention heads (H=4) is incorporated into the output feature layer of the Bi-LSTM to adaptively calculate weights for different features and optimize the parameters of the KF. The simulated data tests demonstrate that the MSC-LA-AKF achieves notably higher denoising accuracy than both the finite impulse response (FIR) and wavelet filters, with detailed quantitative comparisons provided in the experimental section. The proposed method is applied to real airborne gravity data, and effectively removes noise signals and enhances the geological interpretation of gravity maps.

## 1. Introduction

Airborne gravity surveying is an efficient remote sensing technique that records the Earth’s gravitational acceleration continuously from a moving platform, enabling rapid mapping of subsurface mass distributions, mineral targets, and geological structures across extensive regions [1,2,3,4]. Compared with ground-based surveys, airborne gravimetry offers substantially greater spatial coverage and operational flexibility, making it indispensable in mineral exploration, geodesy, and regional geological investigation over areas that are otherwise inaccessible. A modern airborne gravimetry system is a tightly integrated multi-sensor platform comprising a gravity sensor that measures specific force (i.e., the combination of gravitational acceleration and platform motion), a Global Navigation Satellite System (GNSS) receiver that provides accurate position and velocity, and an Inertial Measurement Unit (IMU) that records angular velocity and specific force at high sampling rates [5,6]. The fusion of these complementary sensors enables the recovery of the true gravitational signal by removing the kinematic acceleration of the aircraft from the measured specific force.

Despite its advantages, the accuracy of airborne gravity data is fundamentally limited by multi-source, non-stationary noise [5,6]. Instrument noise arises from sensor electronics and manifests as a combination of Gaussian white noise and a long-term random walk drift. Platform dynamic noise is induced by aircraft attitude changes and vibrations, and can be modeled as a second-order Gauss–Markov process with time-varying intensity. Kinematic noise is non-linearly coupled with the carrier acceleration and its rate of change, and its magnitude varies significantly across different flight phases. These heterogeneous noise components are superimposed on the gravity signal in a time-varying and highly entangled manner, rendering the raw measurements unsuitable for direct geological interpretation. High-precision denoising is therefore a prerequisite for all subsequent data processing and geophysical analysis.

Classical filtering methods have been the primary tools for airborne gravity denoising. Finite Impulse Response (FIR) low-pass filters attenuate high-frequency noise via linear convolution with a windowed kernel, effectively suppressing aircraft vibration artifacts while preserving low-frequency gravity signals [7,8,9,10]. Wavelet thresholding filters decompose the signal into multi-scale coefficients and apply soft or hard threshold criteria to suppress noise-dominant coefficients, enabling simultaneous denoising across multiple frequency bands [11,12,13]. While these methods are computationally efficient and easy to implement, they share a fundamental weakness: both rely on manually selected, time-invariant parameters that cannot adapt to the non-stationary and rapidly evolving noise conditions of real airborne surveys [14]. When noise statistics change during flight—due to turbulence, maneuvers, or sensor drift—these fixed-parameter filters either over-smooth geologically meaningful gravity anomalies or leave substantial residual noise in the output.

The Kalman Filter (KF) provides a principled probabilistic framework for recursive state estimation under Gaussian noise, making it well-suited for multi-sensor integration in airborne gravimetry [15,16,17]. By jointly fusing gravity measurements with GNSS-derived positions and velocities and IMU-derived attitudes, the KF yields optimal state estimates that account for the dynamics of both the signal and the measurement system. Yuan et al. [18] demonstrated the effectiveness of KF in real-time joint filtering of gravity and gravity-gradient data. However, the filtering performance critically depends on the accurate specification of the process noise covariance *Q* and observation noise covariance *R*, which are difficult to determine empirically for the time-varying airborne noise environment. Classical adaptive KF approaches address this by statistical estimation: Mehra [19] combined noise statistics with Kalman gain updates; Assa and Plataniotis [20] estimated prediction error covariance from residual samples; Alaaudeen et al. [21] incorporated a fading factor for strong tracking; Coronado et al. [22] minimized a windowed cost function on EKF errors; and Ge et al. [23] updated *Q* from model deviations. Although these methods improve adaptability, their computational complexity and reliance on residual statistics limit performance under rapidly varying noise conditions.

With the rise of deep learning, AI-enhanced KF methods have emerged as a powerful alternative for adaptive filtering [24,25,26,27]. One line of work replaces the KF entirely with end-to-end neural networks [28,29,30], offering strong nonlinear modeling capacity but sacrificing physical interpretability. The other retains the KF structure and uses neural networks to estimate critical parameters or substitute specific modules [31,32,33], yielding more interpretable models and better robustness under non-stationary noise. Nevertheless, these frameworks have predominantly been validated in robotics, navigation, and medical sensing, and do not explicitly account for the noise characteristics driven by aircraft flight dynamics—including attitude-coupled platform vibrations, kinematic interference, and low-frequency baseline drift—that dominate the airborne gravimetry noise environment. This domain gap motivates the development of a purpose-built, deep learning-driven adaptive KF framework specifically tailored to the non-stationary noise structure of airborne gravity measurements.

To address these fundamental challenges in airborne gravity data denoising, we propose MSC-LA-AKF, a deep learning-driven adaptive Kalman filtering framework that integrates three complementary modules for robust noise covariance estimation under non-stationary airborne survey conditions. The framework employs a Multi-Scale CNN (MSC) with five parallel convolutional branches of varying kernel sizes to simultaneously extract gravity signal features at multiple temporal resolutions, capturing both transient noise events and long-term flight-dynamic drift from the composite observation sequence. To model the time-varying noise patterns driven by aircraft dynamics, a two-layer Bidirectional LSTM (Bi-LSTM) followed by a layer-normalized fully-connected projection and a dual-pooling multi-head attention module is developed, enabling the network to capture bidirectional temporal dependencies and adaptively focus on the observation windows most informative for noise covariance estimation. These learned features are fed into two physics-constrained output sub-networks that produce time-varying scaling factors $\alpha_{k}$ and $\beta_{k}$ with hard clamping constraints, ensuring the adaptive covariance matrices $Q_{k}=\alpha_{k}\bar{Q}$ and $R_{k}=\beta_{k}\bar{R}$ remain physically valid throughout the filtering process. The synergy of these three components enables the KF to dynamically track the rapidly evolving noise environment of airborne gravity surveys, achieving superior denoising accuracy while preserving the geologically meaningful signal content required for structural interpretation and mineral exploration.

In summary, the main contributions of this paper are threefold. First, we propose MSC-LA-AKF, a deep learning-driven adaptive Kalman filtering framework specifically designed for non-stationary airborne gravity measurements, which integrates multi-scale spatial feature extraction and temporal attention modeling into the probabilistic filtering process to enable dynamic tracking of rapidly evolving noise statistics during flight. Second, we introduce a multi-resolution feature extraction mechanism combined with a physics-constrained adaptive covariance estimation strategy: the multi-resolution module captures heterogeneous noise components across different temporal scales—effectively disentangling transient vibration artifacts, kinematic interference, and long-term baseline drift—while the physics-constrained output ensures that the learned time-varying scaling factors maintain positive semi-definiteness of the noise covariance matrices, preserving the interpretability and theoretical consistency of the Kalman Filter. Third, extensive experiments on both large-scale simulations and real airborne gravity survey data demonstrate superior denoising accuracy, rapid adaptation to abrupt noise changes, and improved geological interpretability compared with conventional filtering approaches.

## 2. Related Work

### 2.1. Traditional Filtering Methods for Airborne Gravity Data

FIR low-pass filters represent the most established approach to airborne gravity denoising and have been integral to processing pipelines since the early development of the discipline [34,35]. An FIR filter operates by convolving the input signal with a finite-length kernel designed to pass components within a specified frequency band while attenuating those outside it. In airborne gravity applications, the cutoff frequency is set to isolate the low-frequency gravity anomaly content from the higher-frequency noise contributed by aircraft vibrations, structural resonances, and sensor electronics [7,8]. The filter order determines the sharpness of the spectral transition: higher-order designs achieve steeper roll-off but introduce greater computational cost and phase delay. The choice of windowing function—Hamming, Hanning, or Kaiser—governs the trade-off between main-lobe width and side-lobe suppression, directly influencing the balance between noise attenuation and gravity signal fidelity [9,10]. In this study, the Hamming window is adopted for the FIR filter implementation, as it provides a well-balanced compromise between main-lobe width and side-lobe attenuation (first side-lobe level approximately −42 dB), which is particularly suitable for preserving the long-wavelength gravity anomaly content while effectively suppressing high-frequency measurement noise. When properly designed, FIR filters provide a linear phase response that preserves the waveform integrity of gravity anomalies.

Wavelet thresholding offers a multi-resolution alternative by decomposing the signal into localized time-frequency representations across multiple scales, enabling more targeted noise suppression than purely frequency-domain methods. In the standard formulation, a discrete wavelet transform is applied to yield approximation and detail coefficients at each decomposition level; detail coefficients whose magnitudes fall below a threshold are treated as noise and zeroed or shrunk, and the denoised signal is recovered by inverse transform [11,12]. Soft thresholding shrinks all coefficients toward zero to produce smoother reconstructions, while hard thresholding retains or zeros coefficients entirely to better preserve discontinuities. In airborne gravity processing, the decomposition depth and threshold are calibrated to the dominant noise frequency bands, enabling wavelet thresholding to isolate gravity anomalies by selectively suppressing noise-dominant coefficients while retaining those that represent genuine long-wavelength gravity structure [13]. Various wavelet families—including Daubechies, Symlets, and Coiflets—have been investigated, with the choice of mother wavelet significantly influencing the separation between signal and noise components.

Despite their widespread adoption, both FIR and wavelet methods share a fundamental limitation that becomes increasingly problematic under realistic survey conditions [14]. Their performance is governed by parameters—cutoff frequency and filter order for FIR; threshold value and decomposition depth for wavelet—that are selected prior to processing based on assumptions about the noise spectrum and remain fixed throughout. In practice, however, the noise environment of airborne surveys is highly non-stationary: turbulence intensity, aircraft maneuver dynamics, and sensor thermal drift cause noise statistics to vary substantially across different flight phases. A fixed-parameter filter is inevitably a compromise—aggressive settings suppress peak noise but over-smooth gravity anomalies during calmer segments, while conservative settings preserve fine signal features but leave residual noise during turbulent phases. Furthermore, neither method exploits the rich multi-sensor context available from GNSS-derived kinematics and IMU-measured attitudes, which carry direct information about the dominant noise sources. These limitations motivate the adoption of filtering frameworks capable of dynamically adapting to changing noise conditions.

### 2.2. Adaptive Kalman Filtering and AI-Enhanced Methods

The Kalman Filter provides a principled probabilistic framework for recursive state estimation under Gaussian noise, making it well-suited for the multi-sensor integration architecture of airborne gravimetry [15,16,17]. In a standard KF formulation applied to airborne gravity, the system state vector incorporates the gravity signal alongside position, velocity, and attitude components derived from GNSS and IMU; the process model describes the expected dynamics of each state variable, and the observation model links the state to the multi-sensor measurements. By jointly processing all sensor streams within a unified probabilistic framework, the KF naturally accounts for the correlated noise structure of multi-source sensor errors and produces statistically optimal state estimates at each time step. Yuan et al. [18] demonstrated the practical effectiveness of this approach in a real-time joint KF scheme for gravity and gravity-gradient data, showing that the framework can suppress multi-source measurement noise while maintaining the temporal continuity of gravity profiles required for geophysical interpretation. The recursive structure of the KF also makes it amenable to onboard processing during flight, an important advantage for operational survey efficiency.

The primary limitation of the standard KF for airborne gravity processing is its sensitivity to the specification of the process noise covariance *Q* and observation noise covariance *R*. When treated as fixed empirical constants, these parameters are inevitably suboptimal under changing noise conditions, producing either excessive smoothing of gravity anomalies or residual noise in the output. Classical adaptive KF methods address this challenge by estimating noise covariance matrices online from measurement residuals. Mehra [19] established the foundation by combining noise statistics with Kalman gain updates to yield a variable-parameter KF. Assa and Plataniotis [20] extended this by estimating the prediction error covariance in real time from residual sample statistics, eliminating the need for prior noise information. Alaaudeen et al. [21] introduced a fading factor into the KF update equations to implement strong tracking, enabling the filter to respond more rapidly to abrupt state changes. Coronado et al. [22] proposed minimizing a windowed cost function on EKF residuals to iteratively refine the covariance parameters. Ge et al. [23] updated *Q* adaptively based on the discrepancy between model predictions and actual observations, improving tracking under model uncertainty. Although these methods represent meaningful advances, their shared reliance on residual statistics introduces latency in tracking rapid noise changes, and their computational overhead can be prohibitive for real-time onboard filtering.

With the rise of deep learning [36], AI-enhanced KF methods have emerged as a powerful alternative for adaptive parameter estimation [24,25,26,27]. One line of work replaces the KF entirely with end-to-end neural networks [28,29,30], offering strong nonlinear modeling capacity but sacrificing the physical interpretability and formal optimality guarantees of the KF framework. The other line retains the KF structure and uses neural networks to estimate critical parameters or substitute specific modules—such as the noise covariance matrices, measurement model, or Kalman gain [31,32,33]—yielding more interpretable models with better robustness to non-stationary noise and greater compatibility with domain-specific physical constraints. However, these frameworks have predominantly been validated in robotics, navigation, and medical sensing, where the noise structure differs substantially from the multi-source, flight-dynamics-driven noise encountered in airborne gravimetry. In particular, none of these methods explicitly accounts for the attitude-coupled platform vibrations, kinematic acceleration interference, or slow long-wavelength baseline drift that are characteristic of airborne gravity surveys. This gap underscores the need for a deep learning-driven adaptive KF specifically tailored to the noise structure of airborne gravity measurements, which the proposed MSC-LA-AKF framework is designed to address.

## 3. Problem Definition

Let $y_{k}\in R$ denote the raw gravity measurement recorded at discrete time step *k*, which is a superposition of the true gravity signal $g_{k}$ and a composite noise term $n_{k}$:

(1) $$y_{k}=g_{k}+n_{k}.$$

The noise $n_{k}$ is non-stationary and heterogeneous. Instrument noise is modeled as a combination of Gaussian white noise and a random walk process, representing inherent sensor errors and long-term drift. Platform dynamic noise is driven by second-order Gauss–Markov processes that capture interference from aircraft attitude changes and vibrations. Kinematics-related noise is non-linearly coupled with the carrier acceleration and its rate of change. Together, these three components render $n_{k}$ impossible to characterize with a fixed statistical model.

The airborne gravity processing pipeline integrates position $p_{k}$, velocity $v_{k}$, and attitude $\theta_{k}$ from GNSS and IMU, together with the raw gravity measurement $y_{k}$, to form the state observation vector $z_{k}={\left[y_{k},\;p_{k}^{⊤},\;v_{k}^{⊤},\;\theta_{k}^{⊤}\right]}^{⊤}$. Within the KF framework, the system dynamics are governed by:

(2) $$x_{k}=F_{k-1}x_{k-1}+B_{k}u_{k}+w_{k},\;w_{k}∼N\left(0,Q_{k}\right),$$

(3) $$z_{k}=H_{k}x_{k}+v_{k},\;v_{k}∼N\left(0,R_{k}\right),$$

where $x_{k}$ is the system state vector, $F_{k-1}$ is the state transition matrix, $H_{k}$ is the observation matrix, and $Q_{k}$ and $R_{k}$ are the process and observation noise covariance matrices, respectively. In traditional KF implementations, *Q* and *R* are treated as fixed empirical constants [37,38], which is inadequate for the time-varying noise statistics of airborne surveys and leads to suboptimal filtering—manifesting as either excessive smoothing of valid gravity anomalies or residual noise in the output. The core challenge is therefore to learn a data-driven mapping $f_{\theta }:R^{T\times d}\to R^{+}\times R^{+}$ from a sliding window of *T* consecutive observation vectors $\left\{z_{k-T+1},\ldots ,z_{k}\right\}$ to time-varying scaling factors $\left(\alpha_{k},\beta_{k}\right)$, such that $Q_{k}=\alpha_{k}\bar{Q}$ and $R_{k}=\beta_{k}\bar{R}$, where $\bar{Q}$ and $\bar{R}$ are baseline covariance matrices. These adaptive parameters are then fed into the standard KF recursion to obtain the optimal gravity signal estimate ${\hat{g}}_{k}$ that minimizes $E\left[∥{\hat{g}}_{k}-g_{k}{∥}^{2}\right]$ [39].

## 4. Proposed Method

### 4.1. Overview

**Figure 1:**
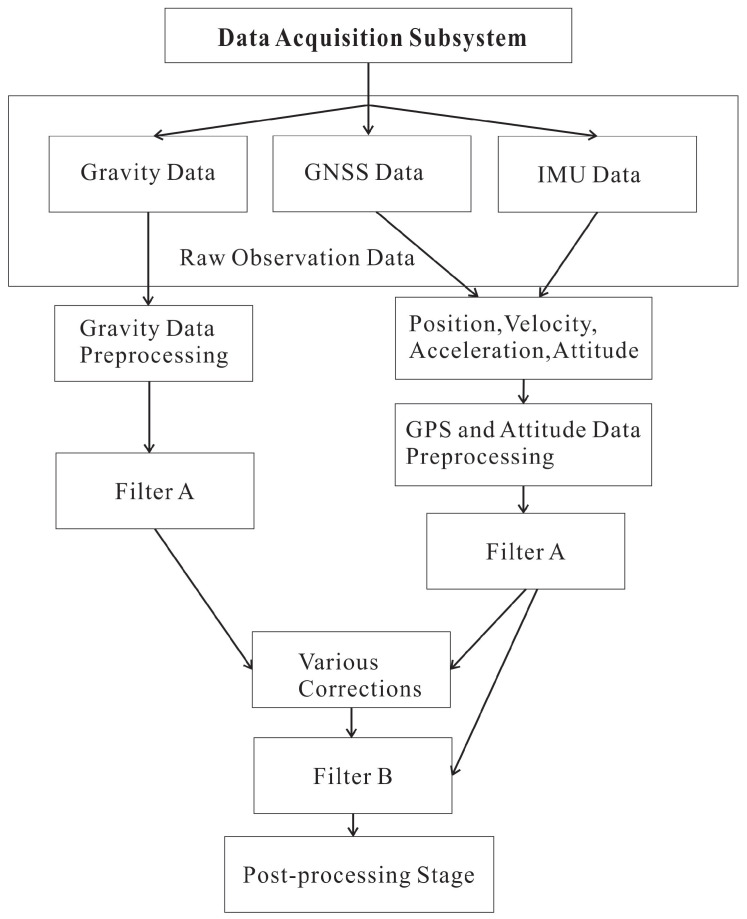
Airborne gravity data processing flow chart. The data acquisition subsystem collects gravity, GNSS, and IMU data. After parallel preprocessing, Filter A pre-filters the signals; subsequent corrections and Filter B yield the final post-processed gravity output.

Airborne gravity data processing first requires calculating the aircraft’s position, velocity, acceleration, and attitude, followed by performing correction and filtering based on this information [40]. The overall processing flow is illustrated in Figure 1, where Filter A pre-filters the raw signal and Filter B performs comprehensive fine filtering of all residual errors to yield the final high-precision output.

**Figure 2:**
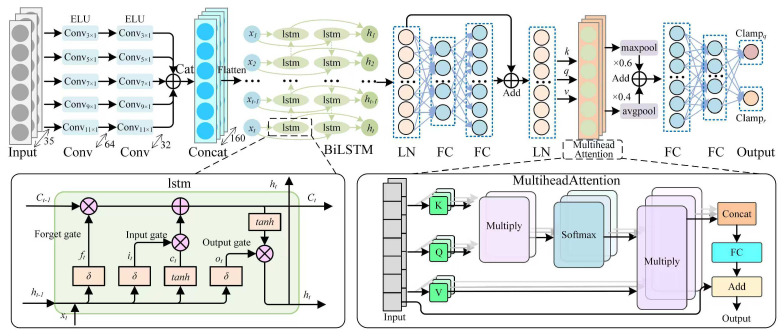
Architecture of the MSC-LA-AKF framework. Five parallel MSC branches (kernel sizes 3–11, ELU activation) extract multi-resolution features that are concatenated (*Cat*) and fed into a two-layer Bi-LSTM. The Bi-LSTM output is layer-normalized (LN), projected by a fully-connected layer (FC), and refined by a dual-pooling multi-head attention module (⊕: addition; ⊗: element-wise multiplication) before two physics-constrained sub-networks output the scaling factors $\alpha_{k}$ (Clamp_*Q*_) and $\beta_{k}$ (Clamp_*R*_). Insets detail the LSTM cell structure ((**left**); δ: sigmoid) and the multi-head attention computation ((**right**); K: key, Q: query, V: value).

The MSC-LA-AKF is designed as an intelligent plug-in for Filter B. It replaces the static empirical *Q* and *R* matrices with time-varying estimates predicted by a deep neural network, while preserving the physical interpretability and recursive structure of the KF. The framework operates as two tightly coupled stages: a deep neural network—comprising five parallel multi-scale CNN branches (kernel sizes 3, 5, 7, 9, 11), a Bi-LSTM layer, a layer-normalized fully-connected projection, and a dual-pooling multi-head attention module—that processes a 35-dimensional sliding-window observation and outputs physically constrained scaling factors $\alpha_{k}$ and $\beta_{k}$; and a standard KF that receives these adaptive factors to compute $Q_{k}=\alpha_{k}\bar{Q}$ and $R_{k}=\beta_{k}\bar{R}$ for recursive gravity signal estimation. The complete architecture is depicted in Figure 2.

### 4.2. Kalman Filter Foundation

The KF provides the recursive estimation backbone of the proposed framework. It operates through a two-step predict-then-update cycle [41]. The prediction step propagates the prior state estimate and covariance:

(4) $${\hat{x}}_{k|k-1}=F_{k-1}{\hat{x}}_{k-1|k-1}+B_{k}u_{k},$$

(5) $$P_{k|k-1}=F_{k-1}P_{k-1|k-1}F_{k-1}^{⊤}+Q_{k}.$$

The update step incorporates the new observation via the Kalman gain:

(6) $$K_{k}=P_{k|k-1}H_{k}^{⊤}{\left(H_{k}P_{k|k-1}H_{k}^{⊤}+R_{k}\right)}^{-1},$$

(7) $${\hat{x}}_{k|k}={\hat{x}}_{k|k-1}+K_{k}\left(z_{k}-H_{k}{\hat{x}}_{k|k-1}\right),$$

(8) $$P_{k|k}=\left(I-K_{k}H_{k}\right)P_{k|k-1}.$$

Here, ${\hat{x}}_{k|k-1}$ is the predicted state, ${\hat{x}}_{k|k}$ is the updated (posterior) state, $P_{k|k-1}$ and $P_{k|k}$ are the corresponding covariance matrices, $F_{k}$ is the state transition matrix, $H_{k}$ is the observation matrix, $B_{k}$ is the control input matrix, $u_{k}$ is the control input, $z_{k}$ is the observation vector, and $K_{k}$ is the Kalman gain. The matrices $Q_{k}$ and $R_{k}$ are supplied by the neural network at each time step, replacing the fixed empirical constants used in conventional implementations.

### 4.3. Encoder Training

#### 4.3.1. Multi-Scale Feature Extraction

Five parallel one-dimensional convolutional branches with kernel sizes $k_{i}\in \left\{3,5,7,9,11\right\}$ (i=1,…,5) are applied simultaneously to the 35-dimensional input observation window $Z\in R^{T\times 35}$. Each branch stacks two convolutional layers—the first with 64 filters and the second with 32 filters, both using stride 1—followed by ELU activation. The output of branch *i* is:

(9) $$h_{i}=\mathrm{ELU}\;\left(W_{i}^{\left(2\right)}*\mathrm{ELU}\;\left(W_{i}^{\left(1\right)}*Z\right)\right),$$

where ∗ denotes 1D convolution, $W_{i}^{\left(1\right)}\in R^{64\times k_{i}}$ and $W_{i}^{\left(2\right)}\in R^{32\times 64}$ are the filter weights, and the ELU activation [42] is defined as:

(10) $$\mathrm{ELU}\left(x\right)=\left\{\begin{array}{cc} x, & x\geq 0,\\ \alpha \;\left(e^{x}-1\right), & x<0. \end{array}\right.$$

The outputs of all five branches are concatenated to form a 160-dimensional multi-resolution feature representation:

(11) $$F_{\mathrm{multi}}=\left[h_{1};\;h_{2};\;h_{3};\;h_{4};\;h_{5}\right]\in R^{160},$$

which captures both short-term transient features and long-term trend components simultaneously. The five branch outputs are concatenated along the feature (channel) dimension at each temporal position, yielding a feature map of shape $T^{\prime }\times 160$, where $T^{\prime }$ denotes the output sequence length determined by the convolution stride and zero-padding configuration. This multi-resolution feature map is then flattened along the channel axis and directly forwarded to the two-layer Bi-LSTM network (Section 4.3.2) for bidirectional temporal dependency extraction, serving as the bridge between spatial multi-scale feature encoding and sequential temporal modeling.

#### 4.3.2. Temporal State Feature Extraction

The concatenated feature sequence $F_{\mathrm{multi}}$ is fed into a two-layer Bidirectional Long Short-Term Memory (Bi-LSTM) network with 64 hidden units per direction. At each time step *t*, the LSTM cell [43] updates its internal state through three gating mechanisms:

(12) $$f_{t}=\sigma \;\left(W_{f}\left[h_{t-1},\;x_{t}\right]+b_{f}\right),$$

(13) $$i_{t}=\sigma \;\left(W_{i}\left[h_{t-1},\;x_{t}\right]+b_{i}\right),\;{\tilde{c}}_{t}=\tanh\;\left(W_{c}\left[h_{t-1},\;x_{t}\right]+b_{c}\right),$$

(14) $$c_{t}=f_{t}⊙c_{t-1}+i_{t}⊙{\tilde{c}}_{t},$$

(15) $$o_{t}=\sigma \;\left(W_{o}\left[h_{t-1},\;x_{t}\right]+b_{o}\right),\;h_{t}=o_{t}⊙\tanh\left(c_{t}\right),$$

where σ(·) is the sigmoid function, ⊙ denotes element-wise multiplication, $f_{t}$, $i_{t}$, $o_{t}$ are the forget, input, and output gates, and $c_{t}$ is the cell state. The bidirectional extension [44] processes the sequence in both the forward (${\vec{h}}_{t}$) and backward (${\overset{\leftarrow }{h}}_{t}$) directions; their outputs are concatenated at each step:

(16) $$h_{t}^{\mathrm{bi}}=\left[{\vec{h}}_{t};\;{\overset{\leftarrow }{h}}_{t}\right].$$

This bidirectional modeling is essential for capturing both forward-causal aircraft dynamics and backward-contextual corrections, enabling identification of time-varying noise patterns such as flight-related baseline drift. The Bi-LSTM output is then layer-normalized and projected through a fully-connected layer with 64 neurons and ReLU activation:

(17) $$p_{t}=\mathrm{ReLU}\;\left(W_{\mathrm{fc}}\cdot \mathrm{LN}\;\left(h_{t}^{\mathrm{bi}}\right)+b_{\mathrm{fc}}\right),$$

where LN(·) denotes layer normalization [45], mapping the temporal representation to a fixed-dimensional space before it is passed to the attention module.

#### 4.3.3. Attention Layer

A multi-head attention module [46] with H=4 heads is applied to the projected feature sequence $p_{t}$. Prior to the attention computation, as an original contribution of this work, we introduce a dual-pooling channel recalibration mechanism that produces a compact global context vector by fusing the max-pooled and average-pooled representations of the sequence:

(18) $$f_{\mathrm{att}}=0.6\cdot f_{\max}+0.4\cdot f_{\mathrm{avg}},$$

where $f_{\max}=\max_{t}\left(p_{t}\right)$ and $f_{\mathrm{avg}}=\frac{1}{T}\sum_{t}p_{t}$ capture global peak and mean feature statistics, respectively. This recalibrated vector adaptively highlights the most noise-sensitive temporal positions in the observation sequence. The query Q, key K, and value V matrices are then formed from $f_{\mathrm{att}}$ via learned linear projections, and the scaled dot-product attention for each head *i* is computed as:

(19) $${\mathrm{head}}_{i}=\mathrm{Softmax}\;\left(\frac{QW_{i}^{Q}{\left(KW_{i}^{K}\right)}^{⊤}}{\sqrt{d_{k}}}\right)VW_{i}^{V},$$

where $d_{k}$ is the dimension of the key vectors and $W_{i}^{Q}$, $W_{i}^{K}$, $W_{i}^{V}$ are learnable projection matrices for head *i*. The multi-head outputs are concatenated and linearly projected:

(20) $$\mathrm{MultiHead}\left(Q,K,V\right)=\mathrm{Concat}\left({\mathrm{head}}_{1},\ldots ,{\mathrm{head}}_{H}\right)\;W^{O}.$$

A residual connection and layer normalization are applied to yield the final attended feature:

(21) $$g=\mathrm{LN}\;\left(f_{\mathrm{att}}+\mathrm{MultiHead}\left(Q,K,V\right)\right),$$

allowing the model to selectively focus on the observational windows most informative for noise covariance estimation while suppressing redundant historical information.

#### 4.3.4. Physics-Constrained Parameter Output

The attended feature vector g is passed through two independent fully-connected sub-networks $f_{\alpha }$ and $f_{\beta }$ (32 neurons each) to produce raw estimates of the noise scaling factors. As an original contribution of this work, we introduce a hard clamp operation that constrains each output to a physically valid positive range:

(22) $$\alpha_{k}=\mathrm{clamp}\;\left(f_{\alpha }\left(g\right),\;\epsilon ,\;C_{\max}\right),\;\beta_{k}=\mathrm{clamp}\;\left(f_{\beta }\left(g\right),\;\epsilon ,\;C_{\max}\right),$$

where clamp(x,a,b)=max(a,min(b,x)), ϵ>0 prevents degenerate zero-noise estimates, and $C_{\max}$ prevents filter divergence. The adaptive covariance matrices fed into the KF at each time step are then formed as:

(23) $$Q_{k}=\alpha_{k}\;\bar{Q},\;R_{k}=\beta_{k}\;\bar{R},$$

where $\bar{Q}$ and $\bar{R}$ are fixed baseline matrices. This hard physical constraint ensures that $Q_{k}$ and $R_{k}$ always remain positive semi-definite, regardless of the raw network output. The complete network is trained end-to-end by minimizing the root mean squared error (RMSE) between the KF-filtered gravity estimate and the true signal on the simulated training set:

(24) $$L=\sqrt{\frac{1}{N}\sum_{k=1}^{N}{\left({\hat{g}}_{k}-g_{k}\right)}^{2}},$$

where ${\hat{g}}_{k}$ is the output of the KF recursion driven by the adaptive $Q_{k}$ and $R_{k}$, and $g_{k}$ is the ground-truth gravity signal.

The key structural parameters of the network are summarized in Table 1.

## 5. Experiments

### 5.1. Datasets

The training and evaluation datasets comprise two complementary sources: large-scale, high-fidelity numerical simulation data and real airborne gravity survey data collected over a survey area in China.

The simulated data are constructed following a physics-driven approach in which a comprehensive non-stationary noise model is superimposed on a clean gravity model signal, generating training examples that cover the full diversity of noise conditions encountered in practice. Instrument noise is modeled as a combination of Gaussian white noise and a random walk process to simulate inherent sensor errors and long-term drift. Platform dynamic noise is described by a second-order Gauss–Markov process, capturing interference caused by aircraft attitude changes and vibrations. Kinematics-related noise is non-linearly coupled with the carrier acceleration and its rate of change, simulating the impact of motion errors on the recorded gravity signal. The intensity parameters of all noise components are independently and randomly sampled for each time series from a statistically justified range derived from real survey data, ensuring both dataset diversity and model generalization. In total, 50,000 independent time series were generated and partitioned into training, validation, and test sets in an 8:1:1 ratio.

The real airborne gravity data used in this study were acquired over the Baiyinnuoer mining district in Chifeng City, Inner Mongolia Autonomous Region, China. The survey area is situated in the southern segment of the Greater Khingan Range, within the Huanggang–Ganzhuermiao metallogenic belt along the northern margin of the North China Plate, a region recognized as an important silver–lead–zinc polymetallic metallogenic concentration area with favorable geological conditions for mineralization. The terrain is dominated by medium-to-low mountain landforms with pronounced topographic relief, exhibiting an overall NW–SE elevation gradient from approximately 1800 m in the northwest to 600 m in the southeast. The mountain ridges trend predominantly in the NE direction, consistent with the regional dominant structural orientation. Influenced by the superposition and reworking of three major tectonic domains—the Paleo-Asian Ocean, the Mongolia–Okhotsk Ocean, and the Paleo-Pacific—the survey area is characterized by well-developed fault structures, dominated by NE-trending deep-seated faults accompanied by NW-trending strike-slip faults, creating a complex structural framework that provides favorable conduits for magmatic activity and mineralization. Several large silver polymetallic deposits have been discovered in the area, including the Baiyinnuoer skarn-type deposit and the Shuangjianzishan hydrothermal vein-type deposit. Due to the strong surface dissection and complex topographic conditions, conventional ground gravity surveys are difficult to conduct efficiently; therefore, airborne gravity surveying was adopted for data acquisition.

The airborne gravity measurements were collected using a small fixed-wing aircraft as the survey platform at a flight speed of 180 km/h (50 m/s), a sampling frequency of 0.5 Hz, and a line spacing of 500 m, with a nominal flight altitude of 700 m above ground level. The survey data are processed entirely independently of the training data and serve solely for assessing the real-world applicability and generalization capability of the proposed method.

### 5.2. Experimental Setup

#### 5.2.1. Comparison Methods

The proposed MSC-LA-AKF is benchmarked against two classical baselines: the FIR low-pass filter, which suppresses noise in a fixed frequency band via convolution with a windowed finite impulse response kernel [7,8], and the wavelet threshold filter, which applies a soft-threshold criterion to multi-scale wavelet coefficients for multi-resolution signal separation [11,13]. For the wavelet baseline, the Daubechies-8 (db8) wavelet is employed across five decomposition levels; db8 is selected for its compact support and smooth approximation properties, which are well-suited to representing the relatively smooth long-wavelength gravity anomaly content while isolating high-frequency noise at finer scales. All three methods are evaluated under a unified experimental framework on identical test sets to ensure a consistent and fair comparison.

#### 5.2.2. Evaluation Metrics

For simulated data, where the true gravity signal $g_{k}$ is available, the mean squared error (MSE) measures the average squared deviation between the filtered output ${\hat{g}}_{k}$ and the ground truth over *N* samples:

(25) $$\mathrm{MSE}=\frac{1}{N}\sum_{k=1}^{N}{\left({\hat{g}}_{k}-g_{k}\right)}^{2}.$$

For real airborne data, where the true gravity signal is unknown, the denoising performance is assessed through qualitative comparison of gravity anomaly maps, examining the suppression of flight-direction striping artifacts, the preservation of geological structures, and the overall spatial coherence of the filtered results.

#### 5.2.3. Implementation Details

The MSC-LA-AKF is implemented in PyTorch. All experiments are conducted on a workstation equipped with an Intel Core i9-13900K CPU, 64 GB DDR5 RAM, and a single NVIDIA GeForce RTX 4090 GPU (24 GB VRAM). The software environment consists of Python 3.10, PyTorch 2.1.0, and CUDA 12.1, running on Ubuntu 22.04 LTS. Training employs the AdamW optimizer [47], which extends the standard Adam optimizer by decoupling the $\ell_{2}$ weight decay from the gradient-based update step; this decoupling prevents the weight decay from being rescaled by the adaptive learning rate, leading to more effective regularization and improved generalization compared to $\ell_{2}$-penalized Adam. The initial learning rate of $1\times 10^{-3}$ and the weight decay coefficient of $1\times 10^{-5}$ were selected by a grid search over the ranges $\left[10^{-4},10^{-2}\right]$ and $\left[10^{-6},10^{-4}\right]$, respectively, with the combination minimizing the validation RMSE at the end of training. A batch size of 64 is used throughout.

**Figure 3:**
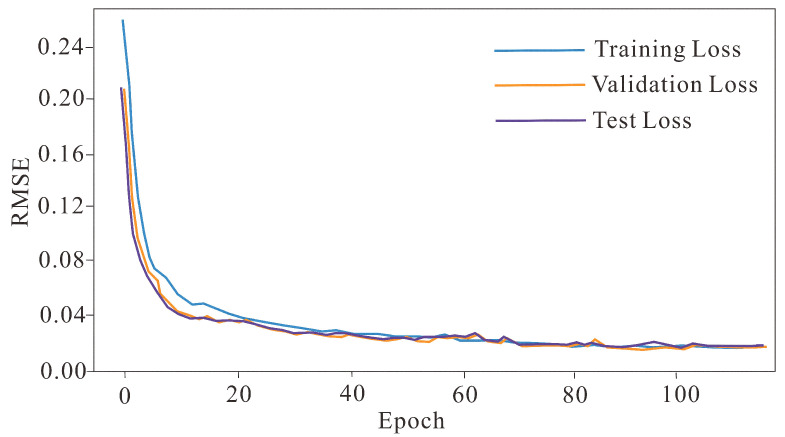
Training, validation and test RMSE curves of MSC-LA-AKF versus training epochs.

A cosine annealing learning rate scheduler gradually reduces the learning rate from its initial value to a minimum of $1\times 10^{-6}$ over each restart cycle, allowing the optimizer to escape shallow local minima while converging to a lower-loss region. An early stopping mechanism monitors the validation RMSE at the end of each epoch and halts training if no improvement greater than $10^{-4}$ is observed over 20 consecutive epochs, retaining the model checkpoint with the lowest validation loss. The training objective is to minimize the RMSE between the KF-filtered output and the true gravity signal. Figure 3 shows the training, validation, and test RMSE curves over approximately 115 epochs. All three curves converge stably to an RMSE of approximately 0.01–0.02, and the close agreement between validation and test RMSE confirms that the model generalizes well without overfitting.

### 5.3. Results and Analysis

#### 5.3.1. Performance Assessments

**Figure 4:**
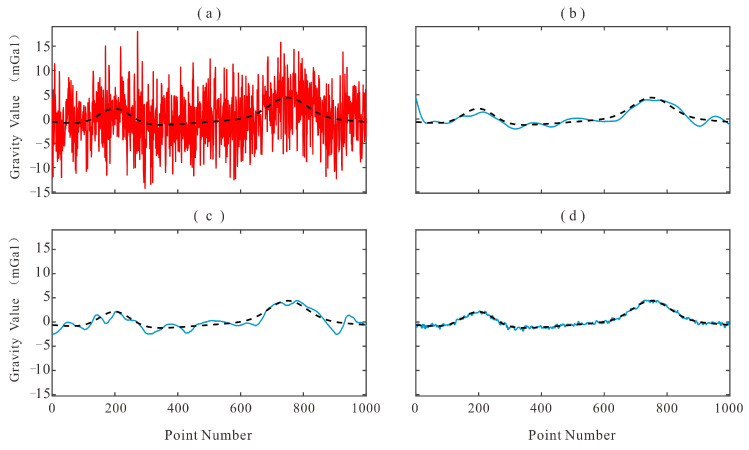
Comparison of filtering results on simulated data: (**a**) noisy measurement (red) and true gravity signal (black dashed); (**b**) FIR-filtered signal (blue) vs. true signal (black dashed); (**c**) Wavelet-filtered signal (blue) vs. true signal (black dashed); (**d**) MSC-LA-AKF-filtered signal (blue) vs. true signal (black dashed).

**Figure 5:**
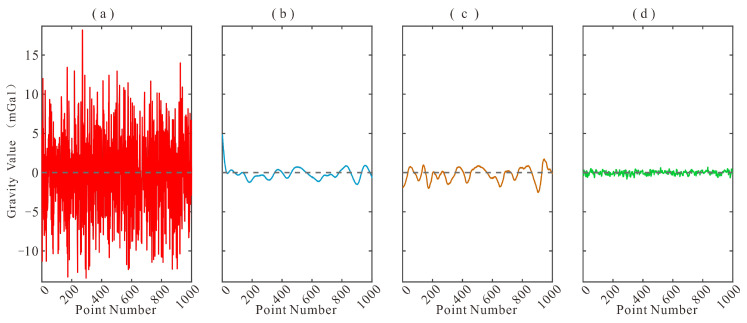
Difference between noisy data and filtered data for each method: (**a**) original noisy signal (red) with zero reference (black dashed); (**b**) residual noise after FIR filtering (blue); (**c**) residual noise after Wavelet filtering (orange); (**d**) residual noise after MSC-LA-AKF filtering (green). The residual noise of MSC-LA-AKF is the smallest and most uniformly distributed, confirming its superior noise suppression capability.

To assess the overall denoising capability of the proposed method, MSC-LA-AKF is compared against the FIR low-pass filter and the wavelet threshold filter on the simulated test set. The visual filtering results are presented in Figure 4, the corresponding residual noise (difference between noisy and filtered data) is shown in Figure 5, and the quantitative results are summarized in Table 2.

A visual inspection of Figure 4 reveals qualitative differences among the three methods. Figure 4a shows the raw noisy measurement superimposed on the true gravity signal, illustrating the severity of the noise contamination. The FIR-filtered output (Figure 4b) closely follows the true signal trend but exhibits residual low-frequency oscillations near high-gradient transitions, a consequence of the fixed cutoff frequency that cannot track non-stationary noise bursts. The wavelet-filtered output (Figure 4c) successfully suppresses high-frequency components but tends to over-smooth local gravity anomaly features, particularly at the peaks and troughs of the signal, because the fixed threshold removes coefficients that carry genuine signal energy at finer scales. In contrast, the MSC-LA-AKF-filtered output (Figure 4d) maintains close agreement with the true signal throughout the time series, preserving sharp local features while simultaneously suppressing broadband noise, reflecting the ability of the adaptive covariance framework to continuously re-tune the KF to the prevailing noise regime. The residual noise plots in Figure 5 further corroborate these observations: the FIR residual (Figure 5b) retains visible low-frequency oscillatory patterns, the wavelet residual (Figure 5c) shows moderate systematic deviations, whereas the MSC-LA-AKF residual (Figure 5d) is the smallest in amplitude and the most uniformly distributed around zero, confirming its superior and more uniform noise suppression across the entire time series.

Quantitatively, as reported in Table 2, MSC-LA-AKF reduces the MSE from 5.4281 mGal to 0.5070 mGal, yielding an improvement rate of 90.66%. This substantially outperforms FIR filtering (MSE after: 1.5001 mGal, IR: 72.36%) and wavelet filtering (MSE after: 1.3127 mGal, IR: 75.82%), representing relative MSE reductions of 66.2% and 61.4% over the two baselines, respectively. The performance advantage of MSC-LA-AKF is attributed to its ability to dynamically adapt the noise covariance parameters based on the multi-modal observation context, in contrast to the time-invariant filtering strategies of FIR and wavelet methods. These results confirm that the deep learning-driven adaptive framework better exploits the intrinsic correlation between multi-source sensor signals and the non-stationary noise environment of airborne gravity surveys.

#### 5.3.2. Ablation Study

**Figure 6:**
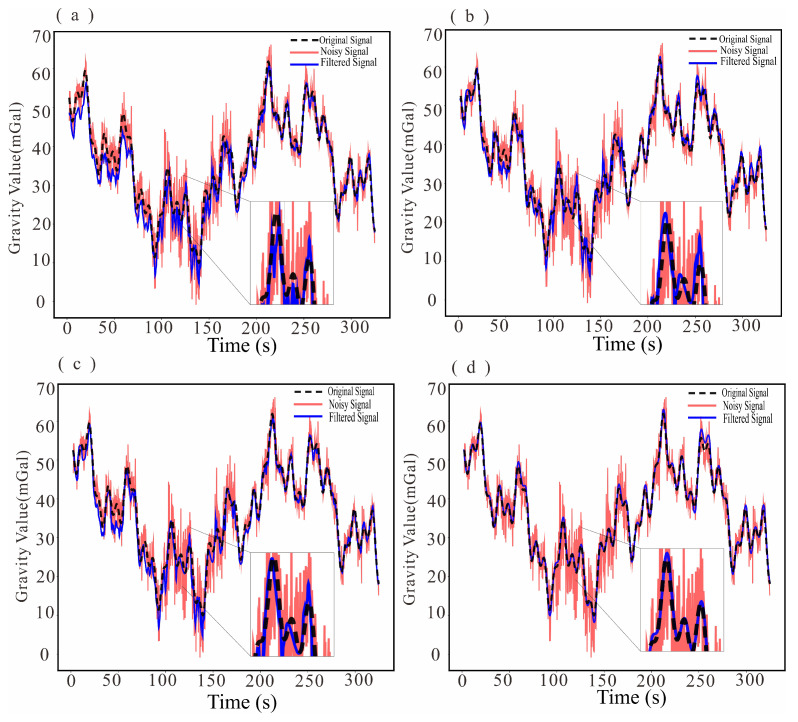
Filtering results in the ablation experiment (each subplot: black = original signal, red = noisy signal, blue = filtered signal): (**a**) Only LSTM model; (**b**) LSTM-Attention; (**c**) Bi-LSTM; (**d**) MSC-LA (proposed).

To quantify the contribution of each architectural component, four progressively augmented model variants are evaluated on the same simulated test set: a unidirectional LSTM baseline (Only LSTM) with no supplementary modules; an LSTM-Attention variant that appends temporal-decay attention to the LSTM output; a Bi-LSTM variant that replaces the unidirectional recurrence with bidirectional modeling while omitting attention; and the full MSC-LA model that unifies multi-scale CNN, Bi-LSTM, and attention into a single end-to-end architecture. The filtering results are shown in Figure 6, and the quantitative results are reported in Table 3.

The visual filtering results in Figure 6 corroborate the quantitative findings. The Only LSTM output (Figure 6a) diverges markedly from the true signal, producing a smoothed trace that fails to track the gravity anomaly shape, which is reflected in the negative improvement rate (IR = −5.57%). This outcome indicates that a unidirectional LSTM, processing only the causal (forward) temporal direction, cannot distinguish between signal and noise components in a system where the noise is correlated with future flight states. Incorporating attention into the unidirectional model (LSTM-Attention, Figure 6b) provides only marginal gain (IR = 4.79%), because the attention mechanism cannot compensate for the fundamental limitation of unidirectional temporal coverage in the absence of multi-scale feature representation.

The introduction of bidirectional modeling (Bi-LSTM, Figure 6c) produces a qualitative leap to IR = 63.38%, confirming that access to both forward-causal and backward-contextual information is critical for estimating the time-varying noise covariance of an airborne gravity system. By processing the observation sequence from both directions simultaneously, the Bi-LSTM can relate the current noise state to both past flight maneuvers and future trajectory constraints, enabling a substantially more accurate separation of gravity signal from dynamic noise. The full MSC-LA model (Figure 6d) achieves the best performance (IR = 69.27%), with a further reduction of approximately 8.7% in MSE relative to the Bi-LSTM-only variant. This incremental gain demonstrates that the multi-scale CNN branches provide complementary information that is not captured by the temporal modeling alone: by simultaneously extracting features at scales corresponding to short-term vibration events (small kernels) and long-term baseline drift (large kernels), the MSC module enriches the feature space available to the Bi-LSTM and attention layers, enabling finer-grained covariance estimation.

#### 5.3.3. Robustness Analysis

**Figure 7:**
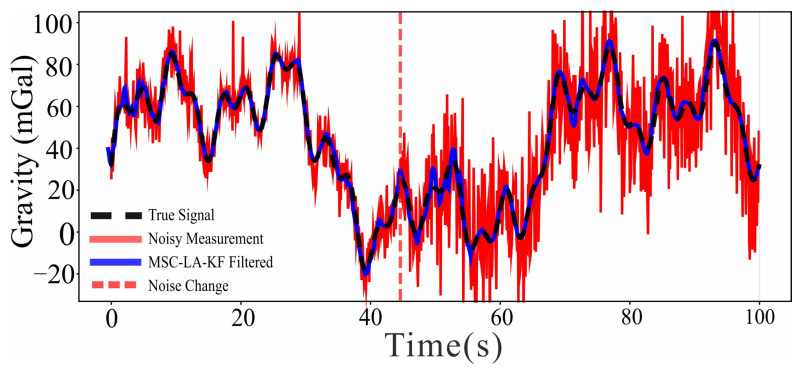
Robustness analysis of MSC-LA-AKF under a 200% noise intensity surge. The pink dashed vertical line marks the noise change point at t≈45 s. Legend: MSC-LA-AKF filtered (black dashed), true signal (blue), noisy measurement (red).

To evaluate stability under adverse conditions, we simulate an abrupt deterioration of the noise environment at the 46th time step of a 100-step segment: Gaussian white noise amplitude is tripled, impulse noise frequency is doubled, and platform dynamic noise power spectral density is increased by 150% (an overall 200% increase in noise intensity). The filtering result is shown in Figure 7.

As shown in Figure 7, the filtered output (black dashed) tracks the true gravity signal (blue) closely prior to the noise surge at t≈45 s. Immediately following the mutation, a brief transient deviation is visible, after which the filtered output rapidly reconverges to the true signal. The MSE rises from 2.3467 mGal before the surge to 3.1679 mGal immediately after (recovery ratio =1.35), and stable filtering performance is fully restored within 1.0 s of the noise change point. Critically, no persistent bias or oscillatory artifact is introduced after the disturbance, demonstrating that the increase in $Q_{k}$ and $R_{k}$ estimated by the network does not destabilize the KF recursion. This behavior is in contrast to classical residual-based adaptive KF methods, which require a sliding window of residuals to estimate the changed noise statistics and therefore exhibit a latency proportional to the window length before recovering accurate covariance estimates. The 1.0 s recovery time of MSC-LA-AKF is well within the temporal resolution required for airborne gravity profiling at typical survey speeds, confirming the practical suitability of the method for real-time or near-real-time onboard processing.

#### 5.3.4. Real Data Processing and Analysis

The proposed MSC-LA-AKF is applied to process all flight lines acquired over the Baiyinnuoer survey area described in Section 5.1. Each flight line is filtered independently using the trained network to produce adaptive noise covariance estimates, and the filtered line data are subsequently interpolated and gridded to generate the two-dimensional gravity anomaly maps presented below.

**Figure 8:**
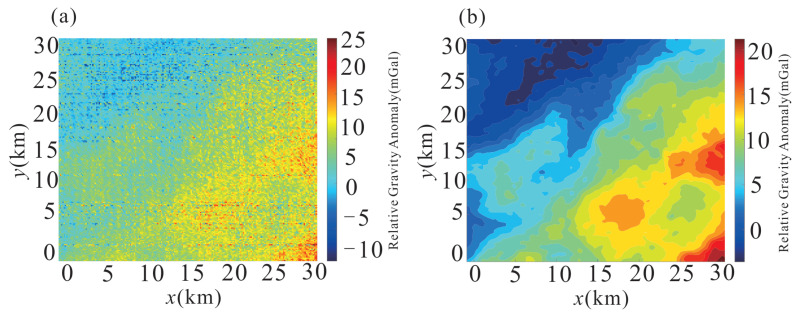
Relative gravity anomaly maps of the Baiyinnuoer survey area: (**a**) after correction and pre-filtering, followed by interpolation and gridding (color scale: −10 to 25 mGal); (**b**) after processing with the proposed MSC-LA-AKF method (color scale: 0–20 mGal).

Figure 8 compares the relative gravity anomaly maps of the Baiyinnuoer survey area under two processing stages. The left panel (Figure 8a) shows the result obtained by applying relevant corrections and pre-filtering to the flight line data, followed by interpolation and gridding; the spatial pattern is dominated by noise-induced striping artifacts oriented along the flight direction, with the color scale spanning −10 to 25 mGal, largely obscuring the underlying geological structure. The right panel (Figure 8b) shows the result after processing with the proposed MSC-LA-AKF method; the color scale narrows to 0–20 mGal and the flight-direction striping is effectively eliminated, revealing a clear gravity anomaly pattern with a prominent high-value zone in the southeastern sector and a low-value zone in the northwestern sector, consistent with the expected density contrast between the exposed basement and the overlying sedimentary cover.

**Figure 9:**
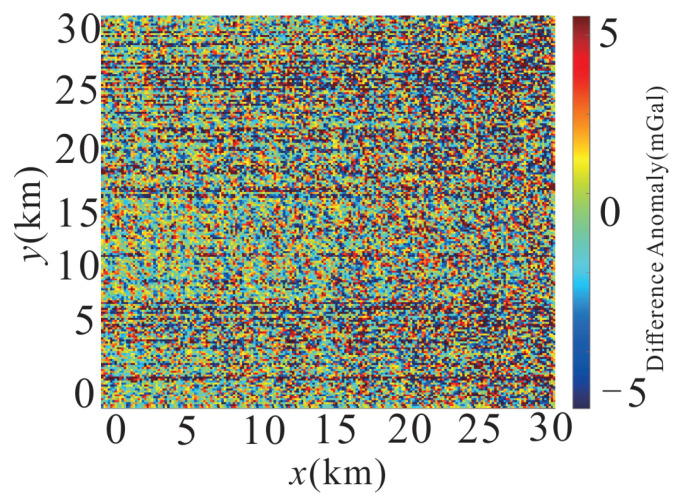
Difference anomaly map (Figure 8a,b) showing the noise component removed by MSC-LA-AKF (color scale: −5 to 5 mGal). The spatially incoherent pattern confirms that the removed component is dominated by measurement noise.

To provide a clearer visualization of the denoising effect, Figure 9 presents the difference map between Figure 8a,b. The difference anomaly ranges from approximately −5 to 5 mGal and is dominated by high-frequency, spatially incoherent fluctuations, confirming that MSC-LA-AKF predominantly removes measurement noise rather than geologically meaningful signal content. The absence of systematic spatial patterns in the difference map further validates that the filtering preserves the structural integrity of the underlying gravity field.

**Figure 10:**
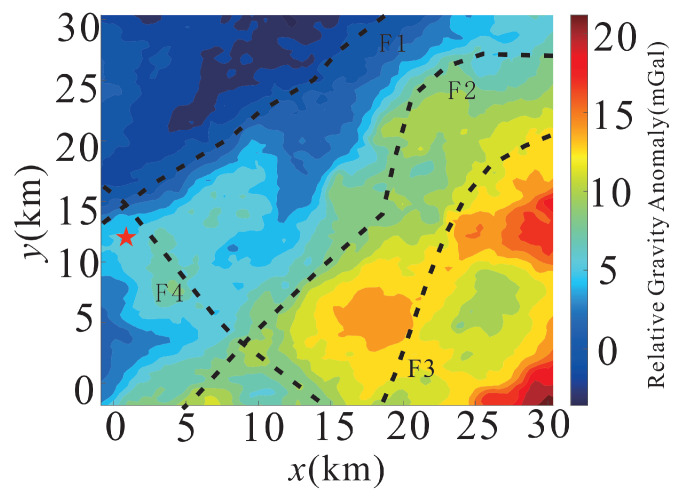
Fault identification results from the MSC-LA-AKF-filtered gravity anomaly map. Black dashed lines indicate identified fault structures: F1, F2, F3 (NE-trending) and F4 (NW-trending). The red star marks the location of the Baiyinnuoer deposit, situated at the intersection of F1 and F4.

To further demonstrate the geological interpretability of the filtered gravity data, boundary detection analysis is applied to the MSC-LA-AKF-filtered gravity anomaly map to identify structural features. The results are presented in Figure 10. The gravity anomaly exhibits a clear NW–SE gradient, with a prominent high-value zone in the southeastern sector and a low-value zone in the northwestern sector. Through boundary identification of the filtered gravity field, three NE-trending fault structures (F1, F2, F3) and one NW-trending fault structure (F4) are delineated. The three NE-trending faults are consistent with the regional Huanggang–Ganzhuermiao fault belt, confirming the reliability of the structural interpretation. Notably, the Baiyinnuoer deposit (red star) is located at the intersection of faults F1 and F4, revealing the close relationship between ore formation and magmatic activity: the fault intersection zone provides favorable conduits for magma intrusion into shallow levels, where contact metasomatic interaction with surrounding rocks creates a favorable environment for skarn-type mineralization. These results demonstrate that MSC-LA-AKF not only suppresses measurement noise but also substantially enhances the spatial resolution and geological interpretability of gravity anomaly maps, providing direct value for subsurface structural mapping and mineral exploration target delineation.

## 6. Conclusions and Future Work

This paper presents the MSC-LA-AKF, a deep learning-driven adaptive Kalman filtering framework for airborne gravity data denoising. The core innovation lies in replacing the fixed empirical noise covariance matrices of the KF with time-varying estimates predicted by a multi-scale CNN–Bi-LSTM–attention network, enabling sub-second adaptation to the rapidly changing noise environment during flight. On simulated data, MSC-LA-AKF achieves an improvement rate of 90.66%, with relative MSE reductions of 66.2% and 61.4% compared to FIR and wavelet filters, respectively. Robustness tests further demonstrate stable recovery within 1.0 s after a 200% noise intensity surge. On real airborne survey data from the Baiyinnuoer mining district, the method effectively eliminates flight-direction striping artifacts from the gravity anomaly maps and substantially enhances the geological interpretability for structural mapping and mineral exploration target delineation.

Several directions merit further investigation. The framework will be extended to jointly process multi-channel gravity gradiometry data, exploiting spatial correlations among gradient tensor components. Model compression and real-time deployment strategies will be explored to enable onboard adaptive filtering during flight operations. Transfer learning approaches will also be investigated to reduce the labeled data requirements when adapting the model to new survey regions with different geological and noise characteristics.

## Figures and Tables

**Table 1:** Key structural parameters of the MSC-LA parameter prediction network.

Network Component	Parameter Name	Parameter Value
Multi-scale Convolutional Branches	Number of Branches	5
	Kernel Sizes	3, 5, 7, 9, 11
	Filters (Layer 1/Layer 2)	64/32
	Stride	1
	Activation Function	ELU
Concatenation Layer	Output Dimension	160 (5×32)
Bi-LSTM Layer	Number of Hidden Units	64
	Number of Layers	2
Fully Connected (FC) Layer	Number of Neurons	64
	Activation Function	ReLU
Attention Module	Number of Heads	4
	Pooling Fusion Weights	0.6 (max) + 0.4 (avg)
Output Sub-network (Process Noise)	Number of Neurons in FC Layer	32
	Output Constraint	Clamp_*Q*_
Output Sub-network (Observation Noise)	Number of Neurons in FC Layer	32
	Output Constraint	Clamp_*R*_

**Table 2:** Quantitative comparison on simulated test data.

Filtering Method	MSE Before (mGal)	MSE After (mGal)	Time (s)
FIR filter	5.4281	1.5001	0.03
Wavelet filter	5.4281	1.3127	0.08
MSC-LA-AKF (Proposed)	5.4281	0.5070	0.42

**Table 3:** Ablation study results.

Network Variant	MSE Before Filtering (mGal)	MSE After Filtering (mGal)
Only LSTM	9.6195	10.1557
LSTM-Attention	9.6195	9.1586
Bi-LSTM	9.6195	3.5229
MSC-LA (Proposed)	9.6195	2.9557

## Data Availability

Data used in this study are publicly available for readers’ reference.
